# A simple method to determine evaporation and compensate for liquid losses in small-scale cell culture systems

**DOI:** 10.1007/s10529-018-2556-x

**Published:** 2018-04-24

**Authors:** Vincent Wiegmann, Cristina Bernal Martinez, Frank Baganz

**Affiliations:** 1 The Advanced Centre for Biochemical Engineering, Department of Biochemical Engineering, University College London, Torrington Place, London, WC1E 7JE UK; 2 Applikon-Biotechnology BV, Heertjeslaan 2, 2629 JG Delft, The Netherlands

**Keywords:** Evaporation, Fed batch, GS-CHO cells, Miniature shaken bioreactor, Reproducibility

## Abstract

**Objectives:**

Establish a method to indirectly measure evaporation in microwell-based cell culture systems and show that the proposed method allows compensating for liquid losses in fed-batch processes.

**Results:**

A correlation between evaporation and the concentration of Na^+^ was found (R^2^ = 0.95) when using the 24-well-based miniature bioreactor system (micro-Matrix) for a batch culture with GS-CHO. Based on these results, a method was developed to counteract evaporation with periodic water additions based on measurements of the Na^+^ concentration. Implementation of this method resulted in a reduction of the relative liquid loss after 15 days of a fed-batch cultivation from 36.7 ± 6.7% without volume corrections to 6.9 ± 6.5% with volume corrections.

**Conclusion:**

A procedure was established to indirectly measure evaporation through a correlation with the level of Na^+^ ions in solution and deriving a simple formula to account for liquid losses.

## Introduction

Biotechnological processes commonly run at elevated temperatures and as a result, the air within the system is typically of high humidity, which can in turn lead to high liquid content in the off-gas. As the vapour leaves the system through the off-gas line, the fill volume of the cultivation vessel will gradually decrease. Not only are the operating conditions directly affected by these volume changes, evaporation can also negatively affect the process through concentrating effects and high osmotic pressures that lead to limitations in cell growth (Silk et al. 2010; Bareither and Pollard 2011; Lattermann and Büchs 2016).

Evaporation issues are inherently less severe in conventional stirred tank reactors (STRs) due to their more favourable surface-to-volume ratio. Furthermore, methods exist to reduce evaporation rates in larger scale STRs. Cooled condensers that return evaporated liquid to the system are standard features of bioreactors and inflowing gases can be humidified before entering the reactor (Doran 2012). Contrastingly, evaporation is a fundamental drawback for both microtitre plates and microbioreactors (Kumar et al. 2004). Not only are the evaporation rates relative to the working volume very high in microtitre plates, the evaporation rates also tend to be higher in the edge and corner wells (Martuza et al. 1976; Deshpande et al. 2004). Efforts to mitigate this phenomenon are based on an increase of humidity in and around the microtitre plate. These include filling the interstices between the wells with sterile water or medium (Girard et al. 2001) or wrapping the plates in damp cloth (Martuza et al. 1976). In addition to these procedural techniques that try to keep evaporation at bay, mechanical sealing solutions have also been developed (Zimmermann et al. 2003). Duetz et al. established an array of well-closure systems that are capable of reducing the evaporation rate in microtitre plates to 20 μL per day (Duetz et al. 2000).

Although well-closures reduce evaporation, inconsistencies in evaporation rates remain a problem for actively aerated systems like microbioreactors in particular, where uneven gas pressures lead to discrepancies in the evaporation rates (Chen et al. 2009). In these systems, evaporation rates are governed by gas flow rates, which in turn may be affected by the culture conditions. Especially in long-term cell culture experiments, it becomes necessary to compensate for the evaporated liquid through periodic additions of sterile distilled water to ensure consistent conditions throughout the experiment. In microtitre plate based formats, the evaporation per well is commonly inferred from the overall weight loss of the plate (Betts et al. 2014). However, this method assumes that the rate of evaporation is the same in all wells, potentially leading to highly variable working volumes and culture conditions. Measuring a concentrating effect of one or more media components can be used as a viable alternative that is less invasive and easily conducted alongside the sample analysis.

This work evaluates the suitability of three potential electrolytes for use as evaporation markers and proposes an inexpensive method to indirectly measure evaporation in small-scale cell culture compartments. This new method is demonstrated as part of an exemplary fed-batch experiment with CHO cells grown in the micro-Matrix microbioreactor.

## Materials and methods

### Pre-culture

Vials of an IgG expressing industrial GS-CHO cell line (Lonza, UK) were thawed and diluted with 49 mL of warmed CD-CHO (Life-Technologies, UK) containing 25 μM MSX. The cells were then expanded for 7 days in a shake flask with a vent cap (250 mL nominal volume, Corning Life Sciences, USA) mounted on an orbital shaker (Sartorius, UK) at 37 °C, 5% CO_2_, and 70% humidity.

### micro-Matrix

The micro-Matrix (Applikon-Biotechnology B.V, The Netherlands) is a micro-bioreactor platform that allows 24 parallel cultivations with individual control of pH, dissolved oxygen (DO) and temperature. The cultivations are carried out in a single-use 24-well cassette with optimum working volume between 2 and 5 mL.

### micro-Matrix cell culture procedure

Offset values for the pH probe calibration were determined by filling each well of the micro-Matrix cassette with 2 mL 1× PBS (Life-Technologies, UK) and mounting the cassette onto the micro-Matrix system. The probes were then left for equilibration without any shaking or further addition of gases. After 1 h, 1 mL was extracted from each well and measured for pH with an offline pH meter (Mettler Toledo, Switzerland). The offset values were then adjusted so that the online measurements matched the offline values.

A suspension with a final concentration of 3 × 10^5^ viable cells mL^−1^ was prepared using the appropriate amount of CD-CHO medium. 3.5 mL of this suspension was filled in each well of the 24-deep square well cassette (Applikon, The Netherlands). The micro-Matrix cassette was covered with the top plate, connected to the gas supply lines and then clamped onto the Optical Thermal Module (OTM) of the micro-Matrix. The set points were specified at pH 7.2, 30% DO, and 37 °C. Down-control of pH was achieved with the addition of CO_2_; the DO was controlled through the addition of O_2_ and N_2_. The shaking speed was set to either 220 or 250 rpm. The pH and DO were both measured in 10 s intervals using optical sensors located at the bottom of each well. The well temperature was controlled for each well with Peltier elements also located at the bottom of each well.

### Fed-batch protocol

Feeding commenced on day 3 of the cultivation and was thereafter repeated every two days. The bolus additions of Efficient Feed B (Life-Technologies, UK) were set to 10% v/v of the initial working volume. Additionally, the cell suspension was spiked with bicarbonate buffer (250 mM Na_2_HCO_3_ and 250 mM NaH_2_CO_3_) set at 2.5% v/v of the initial working volume on day 2, 4, and 5 to adjust the pH upwards during the period of high lactate formation.

### Sampling and analysis

For the batch culture, three wells were sacrificed on each sample day. The cell suspension inside those wells was fully removed and weighed in order to determine the volume loss due to evaporation. All volume-dependent parameters were corrected for loss of volume. In case of the fed-batch culture, sample volumes ranging from 350 to 500 μL were taken from each well upon sampling. The exact volume depends on the analyses that are carried out on a particular day (Table 1). The cell concentration was determined using the Vi-CELL XR (Beckman Coulter, UK) and the remaining cell suspension was centrifuged at 1000×*g* for 5 min. The Bioprofile FLEX analyser (Nova Biomedical, USA) was used to determine the levels of all relevant nutrients, metabolites, and electrolytes. IgG_4_ quantification was conducted using an Agilent 1200 (Agilent Technologies, UK) high-performance liquid chromatography (HPLC) with a 1 mL HiTrap Protein G HP column (GE Healthcare, UK).

**Table 1:** Type of analyses performed and their corresponding sample volumes

	Instrument	Crude sample volume (μL)	Dilution factor
Cell count	Vi-CELL XR	50	10
Nutrients and metabolites	Bioprofile FLEX	166	3
Gases and electrolytes	Bioprofile FLEX	166	3
Titre	Agilent 1200	50	2

## Results and discussion

### Evaluating different markers for evaporation

The initial batch experiment evaluated concentrations of Na^+^, K^+^, and Ca^2+^ as potential markers for evaporation. The choice of this particular set of electrolytes was driven by the measuring capabilities of the available bioanalyser. Further, it is pivotal that the chosen electrolyte has a concentration profile that is generally independent of cellular growth and falls within a suitable concentration range for the bioanalyser to provide accurate measurements.

**Fig. 1:**
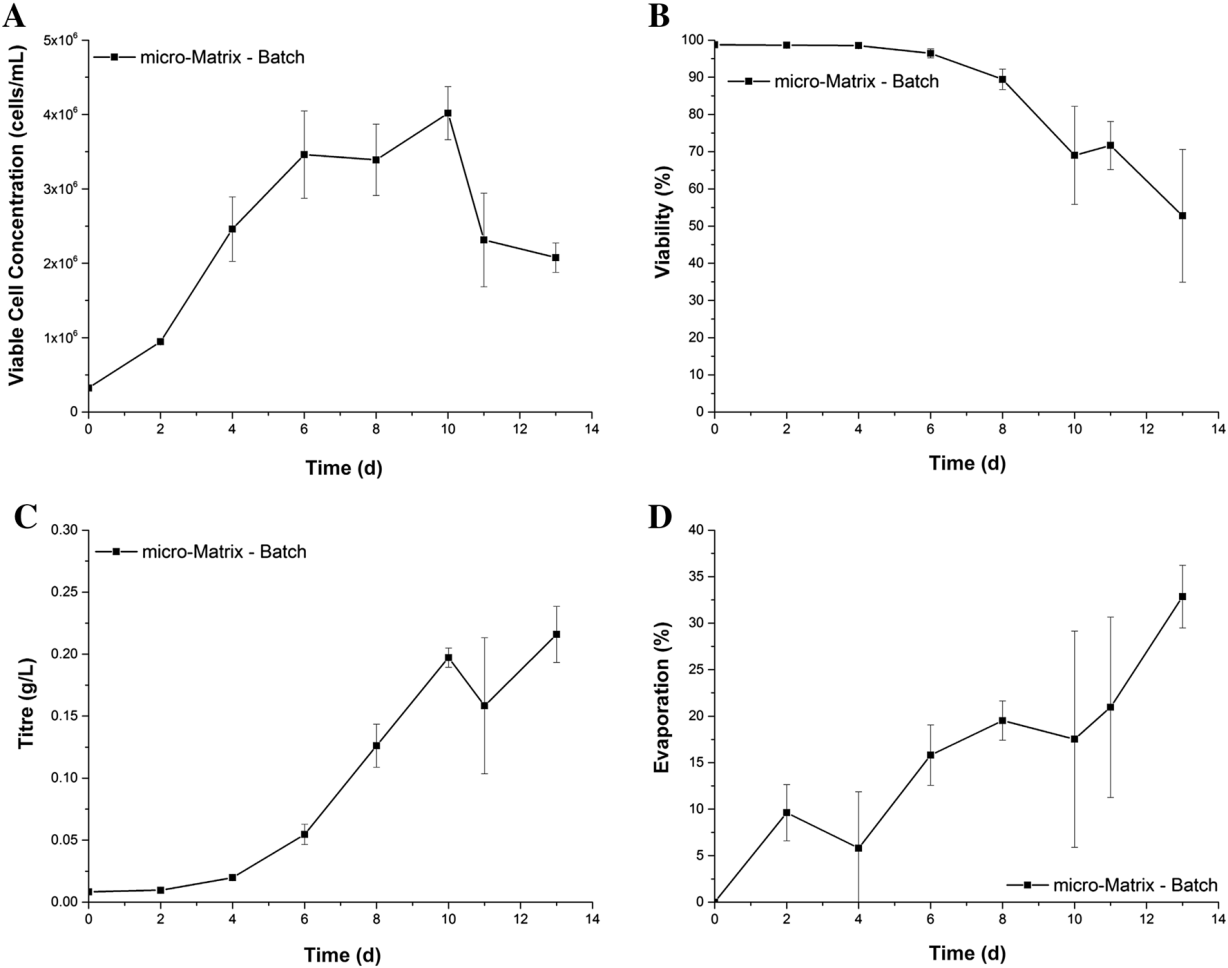
Growth profile (**a**), viability (**b**), titre (**c**), and liquid loss through evaporation (**d**) of GS-CHO cells grown as batch culture in the micro-Matrix at a shaking speed of 220 rpm, a working volume of 3.5 mL, and active control of temperature (37 °C), DO (30%), and pH (7.2). Error bars represent one standard deviation about the mean (n = 3)

The growth and production kinetics in the micro-Matrix shown in Fig. 1 comply with results from previous studies employing the GS-CHO cell line in a similar format (Silk et al. 2010) and therefore, render a typical batch culture. Conversely, the liquid loss exceeded the accepted range early on in the process. The highest degree of evaporation corresponds to the endpoint measurement on day 13 with a liquid loss of 32.9 ± 3.4%. Furthermore, the wide error bars indicate that the evaporation is subject to an extensive degree of variability.

**Fig. 2:**
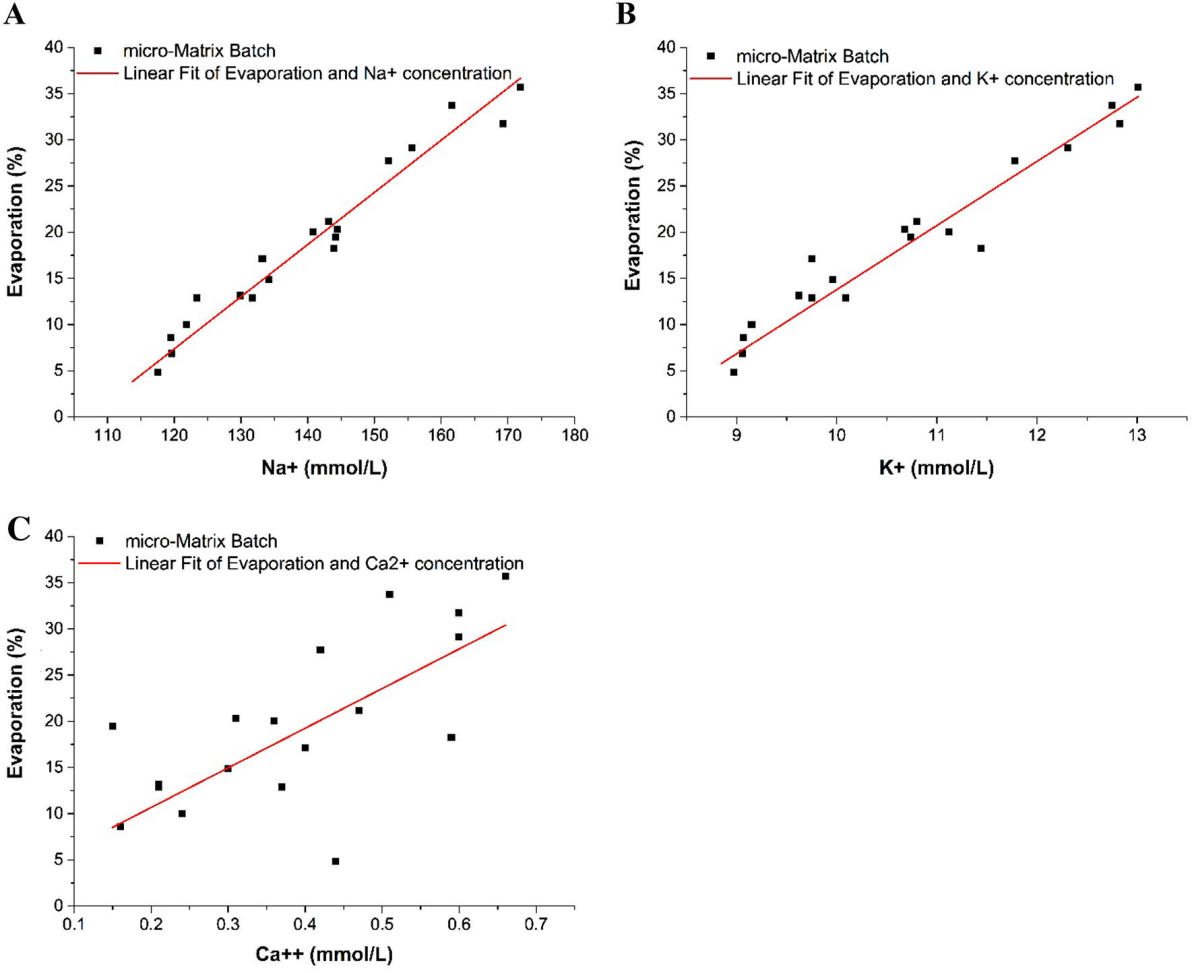
Concentration of the electrolytes Na^+^ (**a**), K^+^ (**b**), and Ca^2+^ (**c**) depending on the relative evaporation (filled square), respective linear correlations (line) during a batch culture with GS-CHO cells grown in the micro-Matrix. The shaking speed was set to 220 rpm at a working volume of 3.5 mL. The DO was controlled at 30% and the pH at 7.2. Na^+^: y = 0.56·x − 60.4, R^2^ = 0.95; K^+^: y = 6.94·x − 55.6, R^2^ = 0.92; Ca^2+^: 42.9·x + 2.1, R^2^ = 0.45. Error bars represent one standard deviation about the mean (n = 3)

Figure 2 shows the relative liquid loss with the corresponding electrolyte concentrations at various time points throughout the batch cultivation. The linearity of the correlation for both K^+^ and Na^+^ suggests that their concentrations remain either minimally affected or entirely unaffected by cellular growth of this specific cell line. The correlation between electrolyte and evaporation is marginally better for Na^+^ (R^2^ = 0.95) compared to K^+^ (R^2^ = 0.92). A correlation is less clear (R^2^ = 0.45) in the case of Ca^2+^; the results are more scattered due to either the cell growth taking an effect on the concentration of Ca^2+^ or because the concentration level is too close to the lower limit of the device’s measurement range (0.1 mmol L^−1^). Because of the slightly improved R^2^ and the concentration levels being well within the measurement range (40–220 mmol L^−1^), Na^+^ was chosen as marker for evaporation.

Based on these findings, a simple correlation between the sodium concentration and evaporation can be established (Eq. 1).

1 $$V_{Evap} = V_{0} - \frac{{V_{0} \cdot \left[ {\text{Na}} \right]_{0} }}{{\left[ {\text{Na}} \right]_{t} }}$$

where *V*_*Evap*_ is the volume of evaporated liquid, *V*_0_ is the initial liquid volume, [Na]_0_ is the initial concentration of sodium ions, and [Na]_t_ is the concentration of sodium at time t.

In case of a fed-batch culture or to account for the addition of other fluids like acid or base, the desired concentration of sodium in the liquid has to be corrected based on the concentration of sodium in the feed medium and the volume of feed medium that is added to the cell suspension. For a system containing the three components, basal medium, buffer, and feed medium, the evaporation can be calculated using Eq. (2).

2 $$V_{Evap} = V_{total} - \frac{{\left[ {\text{Na}} \right]_{medium} \cdot V_{medium} + \left[ {\text{Na}} \right]_{buffer} \cdot V_{buffer} + \left[ {\text{Na}} \right]_{feed} \cdot V_{feed} }}{{\left[ {\text{Na}} \right]_{measured} }}$$

where *V*_total_ is the theoretical volume inside the cultivation chamber at time t.

The choice of electrolytes was largely driven by the measurement capability of the available equipment. Conventional bioanalysers used in an industrial context are often limited to a select few analytes. Although other ions may deliver more accurate estimations of the liquid loss, focusing on the ones that are supported by commonly used equipment renders a more practical approach that can be implemented without additional expenditure.

### Applying the compensation for evaporation in context of a fed-batch cultivation

Fed-batch is to date still the preferred mode of operation in industrial production processes with CHO cells (Pan et al. 2017). By nature, feeding prolongs the duration of the cultivation, potentially giving rise to higher overall evaporation compared to conventional batch cultivations. As a result, the described method to counteract evaporation was tested in the context of a fed-batch operation.

**Fig. 3:**
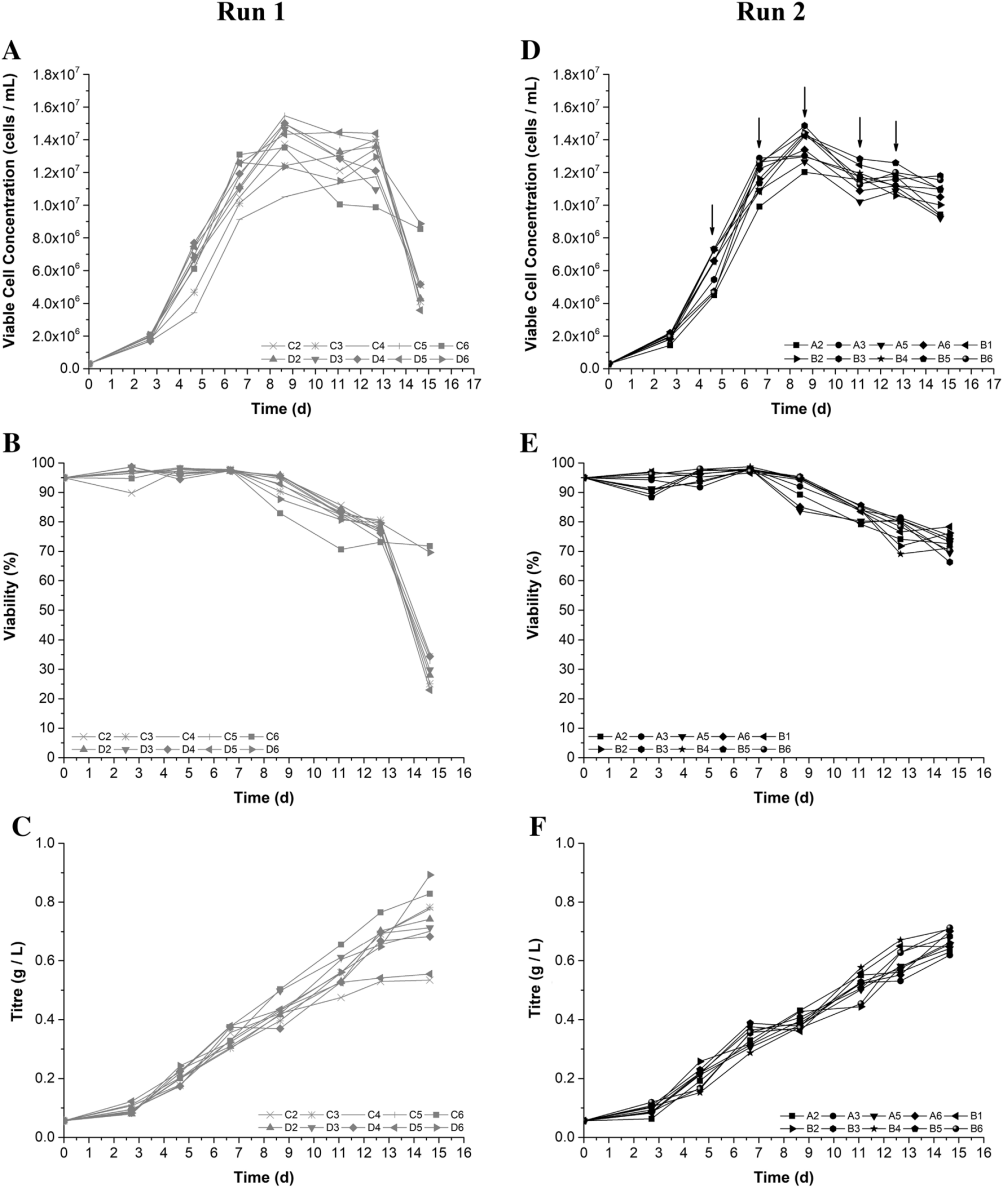
Growth profile (**a**, **d**), viability (**b**, **e**), and titre (**c**, **f**) of GS-CHO cells grown as fed-batch culture in the micro-Matrix at a shaking speed of 280 rpm, a working volume of 3 mL, and active control of temperature (37 °C), DO (30%), and pH (7.2). In run 1 (**a**–**c**) no compensation for evaporation was performed, whereas run 2 (**d**–**f**) was subjected to this procedure five times. Arrows indicate the time points of dH_2_O additions to counteract evaporation based on the sodium level measured

Figure 3 summarises the results of two fed-batch runs performed in the micro-Matrix. In run 1 no volume corrections were performed, in run 2 the procedure was repeated every 2 days from day 5 onwards.

Initially, both cultures progressed in a similar fashion before differences became apparent in later stages of the process. From day 7 onwards, the viable cell density showed higher variability for the wells without volume correction (average CV of 15.5%) compared to the wells in which the evaporated liquid was repeatedly replaced (average CV of 9.5%). The peak viable cell densities were between 12.01 × 10^6^ and 14.86 × 10^6^ cells mL^−1^ for wells with volume correction and 11.75 × 10^6^–15.47 × 10^6^ cells mL^−1^ when liquid loss was not counteracted. Correspondingly, the production kinetics showed a similar increase in spread with final titres ranging from 0.62 to 0.71 g L^−1^ with volume corrections and from 0.53 to 0.89 g L^−1^ without volume corrections. Furthermore, without compensation for the liquid loss, the cell viability decreased prematurely in the majority of the wells.

**Fig. 4:**
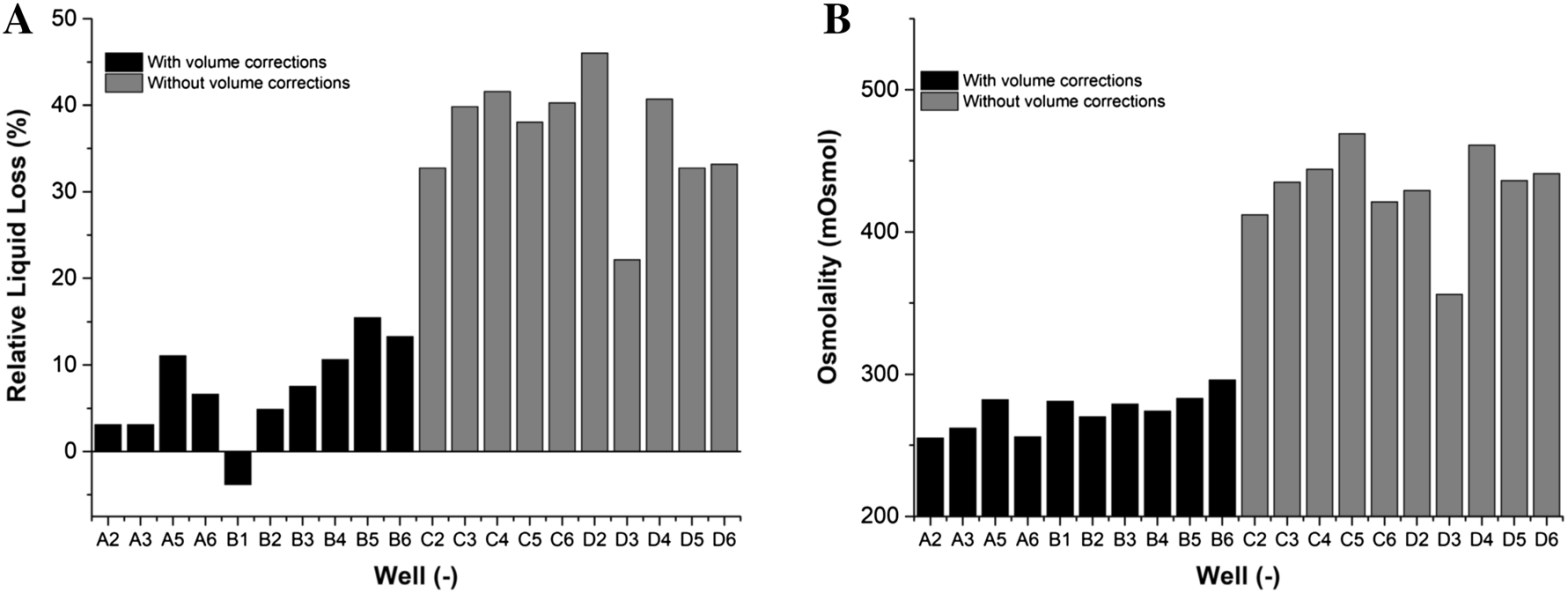
Endpoint values of the relative liquid loss (**a**) and osmolality (**b**) after 15 days of two fed-batch cultivations with GS-CHO cells grown in the micro-Matrix. In run 1 no compensation for evaporation was performed, whereas run 2 was subjected to this procedure five times. The shaking speed for both runs was set to 280 rpm, the working volume was 3 mL, temperature was controlled at 37 °C, DO at 30%, and pH at 7.2

Figure 4 summarises the liquid loss and the osmolality in each well at the end of both runs. Through the repeated volume corrections, the liquid loss could be substantially reduced from 36.7 ± 6.7 to 6.9 ± 6.5% In one case (well B1), the liquid loss was slightly overcompensated, resulting in a negative value. As a consequence of the excessive evaporation, the final osmolalities of the cultures also differed substantially in both conditions. Without volume corrections average values of 273.8 ± 13.1 mOsmol were observed, whereas an average of 430.4 ± 31.2 mOsmol was reached without water additions.

The periodic addition of bolus shots of feed medium successfully increased the longevity of the culture as well as the maximum viable cell count and titre in both runs compared to the batch culture. Without compensation for the liquid loss, the variability of cell growth and product kinetics increased (average CV of 15.5%) and exceeded benchmark values of 10–15% commonly found in the literature (Chaturvedi et al. 2014). Moreover, without volume corrections the process was halted after 15 days of cultivation, which implies a decrease in process performance particularly for long-term cultures. The drop in viability in the cultures without volume correction could be caused by the substantial increase of osmolality, as it has been previously shown that a rise in osmolality can adversely affect growth kinetics (Takagi et al. 2000). Additionally, the liquid loss manifests in a marked difference in working volume and consequently provides a different environment with respect to mixing and mass transfer. In light of such fundamental variations, the benefit of controlling evaporation is clearly demonstrated. Furthermore, a reduced variability can greatly increase the power of screening experiments or early stage cell line characterisation, which are two typical applications for small-scale cell culture systems. As most current bioanalysers allow the quantification of various electrolytes, the presented method is in principle readily implemented into any cell culture process, provided the selected cell line does not affect the electrolyte concentration.

## Conclusion

The concentration of Na^+^ ions can be employed as a marker for evaporation in cell culture applications with GS-CHO. Using the concentration of Na^+^ ions to indirectly measure liquid loss, the relative liquid loss after 15 days of fed-batch cultivation was reduced by approximately 30% compared to cultures without volume corrections. The liquid loss was shown to increase the final osmolality by nearly 60%. Although the growth kinetics followed a similar trend, the lack of volume correction resulted in an increased variability (CV > 15%) and an earlier onset of cell death. Both the increased variability and the change in culture environment in the wells without volume corrections over the course of the cultivation make compelling arguments for the implementation of the described method.

As many of the common bioanalysers are capable of measuring the concentration of sodium, no additional expenditure is required to implement this procedure. Additionally, other electrolytes may be used to infer evaporation in the case that Na^+^ ion measurements are impracticable.
